# Analysis and Optimization of a Synthetic Milkweed Floral Attractant for Mosquitoes

**DOI:** 10.1007/s10886-012-0150-6

**Published:** 2012-06-19

**Authors:** Philip E. Otienoburu, Babak Ebrahimi, P. Larry Phelan, Woodbridge A. Foster

**Affiliations:** 1 Department of Evolution, Ecology & Organismal Biology, The Ohio State University, 318W 12th Avenue, Columbus, OH 43210 USA; 2 Department of Entomology, Ohio Agricultural Research and Development Center, The Ohio State University, 1680 Madison Avenue, Wooster, OH 44691 USA

**Keywords:** *Asclepias syriaca*, *Culex pipiens*, Gas chromatography–mass spectrometry (GC-MS), Milkweed, Nectar feeding, Northern house mosquito, Plant attractants, Subtractive bioassays, Synthetic blends, Asclepidaceae, West Nile virus, Mosquito vector control

## Abstract

A pentane extract of flowers of common milkweed, *Asclepias syriaca* (Asclepiadaceae), elicited significant orientation from both male and female *Culex pipiens* in a dual-port flight olfactometer. Analysis of the extract by gas chromatography–mass spectrometry revealed six major constituents in order of relative abundance: benzaldehyde, (*E*)-β-ocimene, phenylacetaldehyde, benzyl alcohol, nonanal, and (*E*)-2-nonenal. Although not all were collected from the headspace profile of live flowers, a synthetic blend of these six compounds, when presented to mosquitoes in the same levels and proportions that occur in the extract, elicited a response comparable to the extract. Subtractive behavioral bioassays demonstrated that a three-component blend consisting of benzaldehyde, phenylacetaldehyde, and (*E*)-2-nonenal was as attractive as the full blend. These findings suggest the potential use of synthetic floral-odor blends for monitoring or control of both male and female disease-vectoring mosquitoes.

## Introduction

The northern house mosquito, *Culex pipiens* L. (Diptera: Culicidae), is a member of a species complex that includes vectors of lymphatic filariasis and several arboviral diseases, such as St. Louis encephalitis (SLE), Rift Valley fever (RFV), and West Nile virus (WNV). In the northeastern quadrant of the United States, *C. pipiens* recently has become important because of its role in the transmission of WNV. The virus was introduced into New York City in 1999 and is currently the most widespread arboviral disease in the U.S. (CDC, 2010). The blood-feeding pattern of *C. pipiens* (shifting from avian to human feeding within the transmission season) (Edman and Taylor, 1968; Kilpatrick et al., 2006), coupled with its high vector competence (Turell et al., 2005) and ability to carry WNV through the winter, has allowed the virus to spread from its point of origin and maintain transmission throughout the mosquito’s range. Thus, *C. pipiens* is one of the major targets for mosquito vector control and surveillance in the U.S. Current methods of monitoring by abatement districts and health departments include Centers for Disease Control (CDC) light traps and CO_2_-baited traps, which mainly trap blood-seeking females, along with gravid traps, which target gravid females.

Plant-derived attractants have the potential to act as surveillance-trap lures and, when combined with poison-laced sugar solutions (Müller et al., 2010), to control mosquito populations. Mosquitoes are attracted to various plant species, from which they obtain nectar and other juices. Sugar feeding by mosquitoes has a significant influence on dispersal (Hocking, 1953; Magnarelli, 1977) and vectorial capacity (Gary and Foster, 2001; Gu et al., 2011), and, contrary to long-held conjecture, is required by both males and females throughout the adult stage (Downes, 1958; Yuval, 1992; Foster, 1995). Both sexes typically first visit plants soon after emergence. Males then require sugar at frequent intervals to maintain their energy reserves in order to join nightly mating swarms (Yuval et al., 1992). Females take sugar between blood meals, when they are digesting blood, or when they are gravid (Clements, 1999; Foster, 2008). Furthermore, females of most temperate-climate *Culex* and *Anopheles* species enter reproductive diapause in late summer, after which they no longer are attracted to blood hosts, but engage heavily in plant-sugar feeding (Bowen, 1992a).

The potential for plant volatiles to lure mosquitoes has been demonstrated in several laboratory behavioral assays that have used stimuli such as natural plant extracts (Vargo and Foster, 1982; Jepson and Healy, 1988; Mauer and Rowley, 1999) or single floral compounds (Jhumur et al., 2006). In field studies, Bates (1949) reported the successful trapping of anopheline mosquitoes with plant and fruit baits; fruit also proved to be an effective attractant in CDC traps for *C. tarsalis* (Reisen et al., 1986). Sandholm and Price (1962) observed that mosquitoes in the field were attracted to light-colored flowers with distinct fragrances; odor appears to be primarily responsible for long-range attraction, with visual cues playing a role at shorter range (Thorsteinson and Brust, 1962; Healy and Jepson, 1988; Jepson and Healy, 1988). Orientation to commercially obtained floral extracts and honey has been demonstrated for various mosquito species (Thorsteinson and Brust, 1962; Hancock and Foster, 1997; Foster and Takken, 2004).

Schlein and Müller (2008) and Müller et al. (2008, 2010) reported dramatic population reductions of *C. pipiens* and other mosquito species by spraying a fruit-based sugar bait containing insecticide on vegetation surrounding larval habitats. Light-less CDC traps baited with the blossoms of *Tamarix jordanis* were highly effective in trapping *C. pipiens*, and populations were reduced where these blossoms were treated with spinosad insecticide (Schlein and Müller, 2008).

Flower species that elicit the highest attraction probably vary by region. During mid-summer in the U.S., mosquitoes have been observed probing blossoms of common milkweed, *Asclepias syriaca*, at rates disproportionate to their abundance relative to other flowering plants, with one study reporting 54 species occurring in the study area that did not serve as nectar sources (Grimstad and Defoliart, 1974). Another study collected 25 *Aedes vexans* from one milkweed cluster in just 15 min (Sandholm and Price, 1962). The observed preference of insects for milkweed flowers, both day and night, possibly is due to its strong and distinctive fragrance, lighter-colored flowers, and greater nectar production (Sandholm and Price, 1962). Common milkweed is indigenous to eastern and midwestern North America, where its principal native pollinators are mainly large Hymenoptera and Lepidoptera (e.g., Jennersten and Morse, 1991). Mosquitoes are incapable of transferring milkweed pollinia and therefore function as nectar thieves, appearing to co-opt the attractive properties used by the pollinators to locate the flowers (Foster, 1995).

Solvent extracts of common milkweed flowers also are attractive to mosquitoes (Vargo and Foster, 1982; Mauer and Rowley, 1999), confirming that volatile chemicals are at least partly responsible for that attraction. Mauer and Rowley (1999) determined the headspace profile of common milkweed flower to be predominantly 2-phenylethanol and benzyl alcohol; however, they found that a synthetic blend of these two compounds was not attractive to *C. pipiens*. Due to the multiple demonstrations of *C. pipiens* attraction to milkweed, both in the laboratory and the field, we used a solvent extract of the flower as a potential model for synthetic mosquito lures.

## Methods and Materials

### Mosquitoes

Experiments were conducted with *C. pipiens* from a colony established in 2009 from larvae collected near Columbus, OH, USA. Larvae were identified at L4 by siphonal hair tufts (Vinogradova, 2000). Adults were maintained in 41-l clear acrylic cages on a diet of 10 % sucrose, water, and weekly blood meals from the legs of a rooster (ILACUC permit No. 2005A0054). Oviposition water was prepared by soaking grass clippings in aged tap water and allowing fermentation over a 3-d period. Three days after each blood meal, oviposition cups were placed with caged adults, and eggs were collected the following day. Two hundred first-instar larvae were placed into 22.8 × 33.0 cm aluminum pans with 450 ml of aged tap water. The larvae were provided finely ground TetraMin® flakes, increasing the quantity daily from 50 mg for first instars to 500 mg for final instars until pupae appeared on the 8th and 9th day post-hatching. Pupae then were counted and transferred to plastic cups and placed in a 41-l cage supplied with water wicks. Emerging adults were given *ad libitum* access to water, but were deprived of sugar. Experiments were conducted 36 ± 12 h after emergence. The mosquito rearing and maintenance conditions were 27 ± 1 °C, 85 ± 5 % RH, and 16:8 (L:D), with 30-min gradual crepuscular transitions between photophase and scotophase.

### Chemicals

Phenylacetaldehyde (>90 %), benzaldehyde (≥99.5 %, purified by redistillation), nonanal (≥95 %), (*E*)*-2-*nonenal (97 %), and an alkane-standards mixture (C_8_ - C_20_) were purchased from Sigma-Aldrich® (St. Louis, MO, USA). Benzyl alcohol (99.9 %) was purchased from Mallinckrodt Baker, Inc. (Phillipsburg, NJ, USA), and β-Ocimene [(75 % (*E*)-β-ocimene)] was synthesized by CHEMOS GmbH (Regenstauf, Germany). Synthetics were diluted with HPLC-grade *n*-pentane (Fisher Scientific, Pittsburgh, PA, USA).

### Extract Preparation

Flowers of common milkweed, *A. syriaca*, were collected in early summer from The Ohio State University campus in Columbus (40º00′18.95" N, 83º02′47.11" W), placed in an ice cooler, and transported 10-min to the laboratory. Single florets were plucked from the main milkweed umbel and separated from the calyx and other green parts of the flower, weighed, and placed into a 500 ml narrow-mouth glass Erlenmeyer flask. Milkweed florets were submerged in HPLC-grade *n*-pentane (Fisher Scientific) in a 1:8 ratio (w/v) for a total solvent volume of 480 ml and held for 24 h at room temperature, at which time the extract was decanted into 21-ml borosilicate glass vials, capped with Teflon-lined screw caps and stored at -20 °C.

### Headspace Analysis

The volatile profile of milkweed flower was characterized by placing a single floret into a 21-ml borosilicate glass vial with a Teflon-lined rubber septum. Volatiles were allowed to equilibrate in the vial headspace at 30 °C for 10 min before collections were made. Volatiles were collected by using a divinylbenzene/carboxen/polydimethylsiloxane (DVB/CAR/PDMS) (50/30 μm) solid phase micro-extraction (SPME) fiber (Supelco, Bellefonte, PA, USA). This mixed-chemistry fiber provides affinity for chemicals with a far broader range of polarity and volatility than PDMS alone. The fiber was introduced to the vial through the septum and exposed for 30 min. The collected volatiles were analyzed immediately by using an Agilent Technologies 6890 series gas chromatograph interfaced to an Agilent Technologies 5973 quadrupole mass selective detector (GC-MS). The SPME fiber was desorbed at 275 °C for 3 min with a splitless injection onto a Zebron™ ZB-1 ms column (30 m × 0.25 mm, 0.25 μm film thickness) (Phenomenex, Torrance, CA, USA). The oven temperature was held at 25 °C for 3 min, then ramped at 15 °C/min to 250 °C, where it was held for 2 min. The carrier gas was helium at a flow rate of 1 ml/min. The mass selective detector was operated in EI mode at 70 eV, scanning 19-350 *m/z* with a quadrupole temperature of 180 °C and source temperature of 240 °C.

### Extract Analysis

**Fig. 1:**
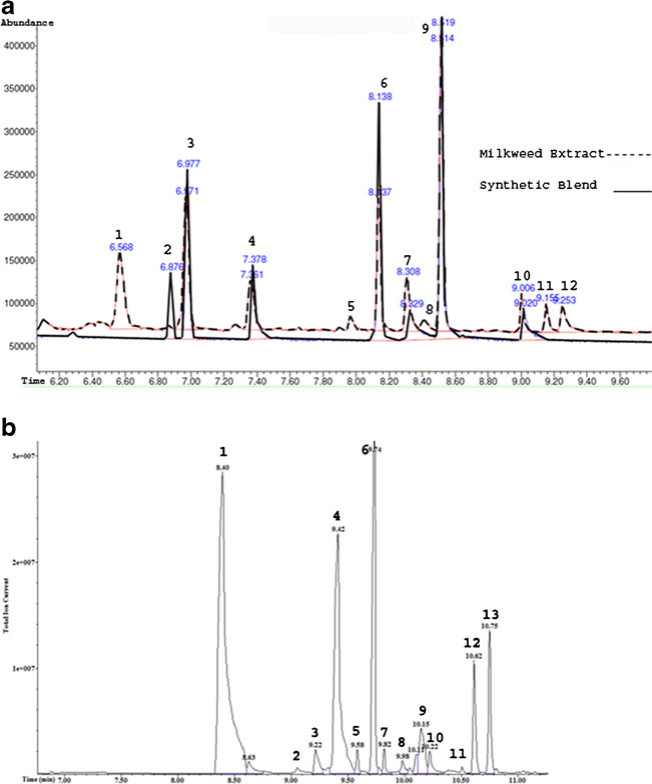
Total ion chromatograms (**a**) of florets of milkweed, *Asclepias syriaca*, pentane extract (broken line), and a synthetic blend (solid line) used to assay upwind attraction of *Culex pipiens* (Peak # 1. unknown, 2. (*Z*)-β-ocimene, 3. (*E*)-β-ocimene, 4. benzaldehyde, 5. unknown, 6. nonanal, 7. benzyl alcohol, 8. unknown, 9. phenylacetaldehyde, 10. (*E*)-2-nonenal, 11. 2,6-nonadienal, and 12. phenylethanol; column = Zebron™ ZB-50) and total ion chromatogram (**b**) of the headspace profile of a single *A. syraica* floret captured on a divinylbenzene/carboxen/polydimethylsiloxane SPME fiber (Peak # 1. benzaldehyde, 2. myrcene, 3. unknown monoterpene, 4. phenylacetaldehyde, 5. (*Z*)-β-ocimene, 6. (*E*)-β-ocimene, 7. γ-terpinene, 8. unknown monoterpene, 9. dimethylstyrene, 10. unknown monoterpene, 11. unknown monoterpene, 12. (*E,Z*)-alloocimene, and 13. (*E,E*)-alloocimene; column = Zebron™ ZB-1)

To guide the construction of a synthetic floral blend, pentane extracts of milkweed florets were analyzed by GC-MS on a Zebron™ ZB-50 column (15 m × 0.25 mm, 0.25 μm film thickness) (Phenomenex), with a 1-μl splitless injection at 210 °C. The temperature program began at 25 °C for 3 min and increased at 13 °C/min to 200 °C, followed by a 25 °C/min increase to 280 °C to remove high-boiling lipids. Other parameters were as described above. Peak identities were validated by re-injection on a Finnegan Trace GC/MS (Thermo Fisher Scientific Inc., Waltham, MA, USA), with a 1:10 split at 180 °C and a Rtx®-5MS column (30 m × 0.25 mm, 0.25 μm film thickness) (Restek, Bellefonte, PA, USA). The helium flow was 1 ml/min, and temperature program was 40 °C for 1 min, ramped at 15 °C/min to 275 °C, with a 2-min final hold time. Retention indices (RI) of compounds were determined on both columns relative to *n*-alkanes (C_8_–C_20_). Components of floral headspace and extract were identified by matching their mass spectra to NIST/EPA/NIH Mass Spectral Library 2005 and comparison of retention indices to published values (Adams, 1995). Identity of major components was confirmed by comparison of spectra and retention times to authentic standards. Peak purity was determined by using AMDIS deconvolution software (NIST, Gaithersburg, MD, USA) to assure the absence of any co-eluting components. A synthetic mimic of the extract was constructed by blending authentic standards of the major components identified in the extract. Quantities of each component were individually adjusted until the overall concentration and component proportions closely approximated those of the floral extract (Fig. 1**a**).

### Behavioral Assays

All behavioral assays were conducted in a clear acrylic dual-port flight olfactometer, modified from Hancock and Foster (1993), with three main parts: an introduction/release chamber, flight chamber, and trapping ports. The introduction/release chamber was located at the downwind end, measuring 30 × 40 cm wide and 30 cm long. A sliding gate separated the release chamber from the main flight chamber (30 × 40 cm wide, 90 cm long), which had two cylindrical glass jar trapping ports (15 cm long by 7 cm diameter) on its upwind end. Ports were fitted with borosilicate glass funnels, with the wide end opening into the flight chamber and the narrower (3 mm diam) end pointing into a glass jar. Thus, mosquitoes entering the wide end of the funnel from the flight chamber were channeled into the jar, where they were retained. Trapping ports were located 11 cm above the flight chamber floor and were separated by 21 cm. The funnel and glass jar were held together by Parafilm^®^ sealing film (Pechiney Plastic Company, Menasha, WI, USA), which was replaced between treatments. We recognized the pitfalls associated with the use of Parafilm^®^ in solvent-based behavioral assays (Millar and Haynes, 1998), and we ensured that this sealant would not come in contact with any chemicals in the olfactometer. A 7.5-mm diam hole in the upwind end of each jar allowed the introduction of purified/humidified air after it had passed from an oil-free air pump through an activated carbon canister, and then through a water column. Air flow was maintained at 50 ml/s into each port, providing a velocity of 72 mm/s in the center of the flight chamber. A black cotton cloth, covering a dampened layer of cotton wool, covered the entire floor of the flight chamber, to maintain a level of humidity similar to that within the choice ports. Data loggers (HOBO®, Onset Computer Corporation, Bourne, MA, USA) recorded temperature and humidity in the olfactometer, which was 25.0-27.5 °C and 75-95 % RH. An exhaust duct, connected at the downwind end of the release chamber, directed the effluent air out of the building through an exhaust hood. To test for positional bias of the ports, mosquitoes were released into the chamber after either baiting both ports with honey or leaving both empty. Mosquitoes were divided equally between left and right ports when baited with honey and were found rarely in ports when both were empty.

Response to whole milkweed flowers was determined in the flight olfactometer with 2 g of florets placed in an aluminum weighing boat in one of the trapping jars, and compared with an empty control jar. Extracts and synthetic chemicals were applied to 15-cm long cotton wicks (TIDI Products, Neenah, WI, USA) and compared to a pentane control. The positions of the treatment and control ports were alternated between bioassays as a further safeguard against positional bias. The olfactometer parts were cleaned with 70 % ethanol followed by water after each experiment; gloves were used at all times to avoid contamination with human-related kairomones. The 16:8 (L:D) light cycle used for mosquito rearing was maintained in the bioassay room. At 2 h prior to scotophase, approximately 200-300 mosquitoes of both sexes in similar numbers were released through a sleeve connection, directly from an acrylic plastic cage (30 × 30 × 30 cm) into the holding/release chamber, where they were held for 15 min to acclimatize before the release gate was opened. After 12 h, the numbers of mosquitoes in the treatment port, the control port, and remaining in the flight chamber were recorded. Use of a 12-h period captured the bimodal nocturnal-crepuscular sugar-feeding rhythm of *C. pipiens*, which includes some sugar feeding throughout the night (Yee and Foster, 1992).

Subtractive bioassays were conducted on the synthetic blend to determine the relative activity of the identified components. The full six-component blend was presented in one of the paired olfactometer ports alongside a reducted five-compound blend, in which one of the components was removed. Each reducted blend was tested 4-6 times. After establishing the significance of each chemical on mosquito attraction, we compared the minimal attractive blend against the full six-component blend. This was followed by a dose–response study of the minimal blend to determine the optimal concentration for response. Doses were presented in a randomized block design (*N* = 5), with percentage response calculated as the number in the synthetic blend port minus the number in the control port and expressed relative to the number of mosquitoes released.

### Video Recording

Mosquito flight behavior was recorded by using three infrared RCA closed-circuit video cameras (Lancaster, PA, USA) onto which was mounted a TC1824B wide-angle ES 25 mm 1:1.4 lens for flight-chamber recordings or a TC1874C ES 75 mm 1:1.8 lens for each of the choice ports. The cameras were controlled in series with a Burle Security Products TC8108 (Lancaster, PA, USA) eight-channel switcher, with video output to two RCA TC1109 video monitors. Video output was recorded with an Emerson EWV404 VCR (Parsippany, NJ, USA) onto VHS tape in time-lapse mode, 20 s each min.

### Statistical Analysis

Mosquito response in the flight olfactometer was analyzed by comparing numbers caught in the two ports by a goodness-of-fit *chi-squared* test with SPSS v. 17 (SPSS Inc, Chicago, IL, USA). The threshold for significance was *α* = 0.05. In the dose–response experiment, a regression model of mosquito response to log-transformed blend dose was determined by using the Fitted Line Plot module of Minitab v. 16 (Minitab Inc., State College, PA, USA).

## Results

### Chemical Identification

Six compounds comprised >90 % of the relative abundance of the milkweed floret components in the pentane extract: (*E*)-β-ocimene, benzaldehyde, nonanal, benzyl alcohol, phenylacetaldehyde, and (*E*)-2-nonenal (Fig. 1a, Table 1). The volatile profile collected by DVB/CAR/PDMS SPME directly from fresh florets was dominated by three of these compounds: (*E*)-β-ocimene, benzaldehyde, and phenylacetaldehyde (>75 % of the total), but there were also substantial differences compared to the pentane extract (Fig. 1b). Most notably, (*E,Z*)- and (*E,E*)-alloocimene were collected from the flower headspace, but were almost completely absent from the extract, whereas nonanal, a major constituent of floret extract, was missing in the headspace sample. These disparities can be explained partly by variation in volatility among the components, but also likely reflect differences between the chemical composition in flower tissue and what is released. The extract also contained higher-boiling hydrocarbons and fatty acids, but due to their low volatility, they were not included in behavioral studies. Based on the quantities of synthetic standards used to mimic the extract GC profile (Table 1), the total floral concentration of the six major volatiles was estimated at 32 μg/g floret fresh weight.

**Table 1:** Major volatile constituents of a pentane extract of the common milkweed, *Asclepias syriaca*, as determined by gas chromatography–mass spectrometry, and quantities used in a synthetic mimic

COMPOUND^a^	Relative retention index^b^	Diagnostic EI-MS fragment ions (% intensity)	Synthetic blend (μg/ml)
(*E*)-β-Ocimene	1124	77(36), 79(42), 91(53), 93(100)	6.14^c^
Benzaldehyde	1155	51(54), 77(100), 105(88), 106(88)	3.27
Nonanal	1220	29(66), 41(100), 56(61), 57(94)	4.14
Benzyl alcohol	1236	77(85), 79(100), 107(59), 108(75)	4.58
Phenylacetaldehyde	1257	65(22), 91(100), 92(24), 120(16)	11.25
(*E*)-2-Nonenal	1304	29(56), 41(100), 55(80), 70(58)	2.12

^a^Identity established by comparison to authentic standard of each compound

^b^GC retention times relative to *n*-alkane standards on a Phenomenex ZB-50 column phase

^c^Synthetic β-ocimene contained a 3:1 (*E:Z*) isomer mixture

### Flight Olfactometer Response

Infrared video recordings showed *C. pipiens* in the flight chamber engaged in a zigzagging flight that was directed upwind towards ports baited with florets, extracts, or synthetic blends. The angle of the turns progressively decreased as the mosquitoes approached the target. In most cases, mosquitoes flew into the ports, but in a few cases they landed on the entrance of the port and walked in. There was no observed oriented flight towards the control port.

**Fig. 2:**
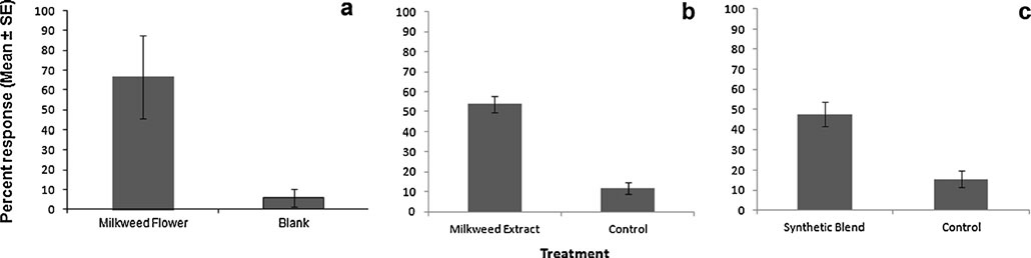
Percentage of *Culex pipiens* flying upwind in a dual-port flight olfactometer to: **a** whole milkweed, *Asclepias syriaca*, florets; **b** pentane extract of *A. syriaca* florets; and (**c)** synthetic *A. syriaca* floret blend compared against a water **a** or solvent (**b, c**) control. *** *P* < 0.001 by *chi-squared* test

In the first behavioral assay, mosquitoes showed a significant response to whole milkweed florets, where 67 % of released mosquitoes were captured in the floret-baited port, compared to only 6 % in the control port (Fig. 2a; *χ* ^*2*^ *=* 515.02 *df* = 1, *P* < 0.001). Despite the chemical differences measured between the headspace and extract profiles of milkweed florets, mosquitoes showed a similarly strong response to a pentane extract of milkweed florets (52 %) compared to pentane alone (11 %) (Fig. 2b; *χ* ^*2*^ = 61.44*, df* = 1, *P* < 0.001). Moreover, mosquitoes were observed in video recordings to probe the extract-treated cotton wick during the early scotophase and early photophase, suggesting that the extract also stimulated a feeding response. This behavior was never observed on control wicks.

Based on the positive response to the milkweed floret extract, a six-component synthetic blend was formulated to simulate the concentrations and relative proportions of major constituents of the pentane extract (Fig. 1a). One discrepancy in the GC profiles was caused by the presence of the (*Z*)-isomer in synthetic (*E*)-β-ocimene, resulting in a synthetic blend that exceeded the levels of this isomer in the floret extract. In the olfactometer, the synthetic floret blend performed similarly to the natural extract, with 48 % of released mosquitoes trapped, compared to 16 % for the control (Fig. 2c; *χ* ^*2*^ *=* 120.6, *df* = 1, *P* < 0.001). Again, video-recordings showed a directed upwind flight response to ports containing the synthetic blend, accompanied by vigorous probing of the treated wicks, but not to the controls. Males and females responded to milkweed floret odors in similar numbers in all three experiments as the sex ratio of mosquitoes captured in either the treatment or control ports did not deviate significantly from 1:1.

**Fig. 3:**
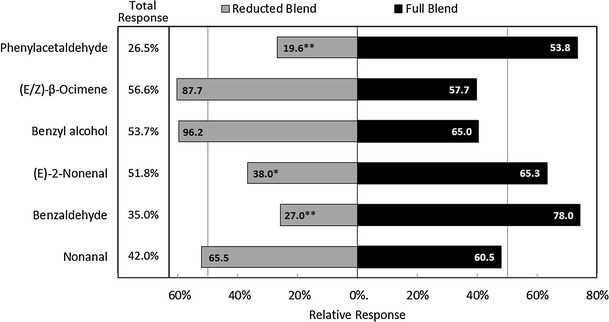
Choice response of *Culex pipiens* in a dual-port flight olfactometer to a six-component (full) synthetic blend of milkweed, *Asclepias syriaca,* floret volatiles compared to blends in which each component has been removed individually (reducted). Total response indicates the percentage of *C. pipiens* released that were captured by one of the blends (no significant differences by 1-way ANOVA). Relative response shows the proportional response to the reducted (gray bars) and full (black bars) blends. Numbers in bars indicate the mean number of *C. pipiens* responding (*N* = 4–6). Asterisks denote significant differences in mean response to reducted *versus* full blend by chi-squared test (* *P* < 0.05, ** *P* < 0.005)

**Fig. 4:**
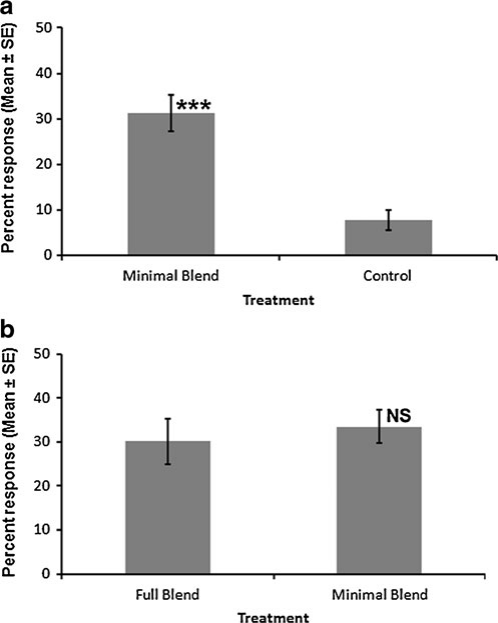
**a** Percentage of *Culex pipiens* flying upwind in a dual-choice flight olfactometer in response to a three-component blend, consisting of benzaldehyde, (*E*)*-2-*nonenal, and phenylacetaldehyde; *** *P* < 0.001 by *chi-squared* test. **b** Percentage of *C. pipiens* flying upwind in response to the full (six-component) and minimal (three-component) synthetic blends of *A. syriaca* floral odor. NS = no significant difference

The subtractive bioassay of the synthetic blend indicated a significant role for three compounds in *C. pipiens* response to milkweed: benzaldehyde (*χ* ^*2*^ *=* 13.22, *df* = 1, *P* < 0.001), phenylacetaldehyde (*χ* ^*2*^ *=* 8.25, *df* = 1, *P* = 0.004), and (*E*)*-2-*nonenal (*χ* ^*2*^ *=* 3.62, *df* = 1, *P* = 0.05) (Fig. 3). When any of these compounds was removed, mosquitoes showed a significant preference for the full blend over the reducted blend. A nearly significant preference was seen for blends missing either β-ocimene (*P* = 0.078) or benzyl alcohol (*P* = 0.082) compared to the full blend. The activity of benzaldehyde, phenylacetaldehyde, and (*E*)*-2-*nonenal was confirmed when the three were combined: the three-component blend captured 31 % of mosquitoes compared to only 7 % for the control (Fig. 4a; *χ* ^*2*^ *=* 196.56, *df* = 1, *P* < 0.001) and was as active as the full blend (Fig. 4b **,** 4; *χ* ^*2*^ = 3.02, *df* = 1, *P* = 0.082).

**Fig. 5:**
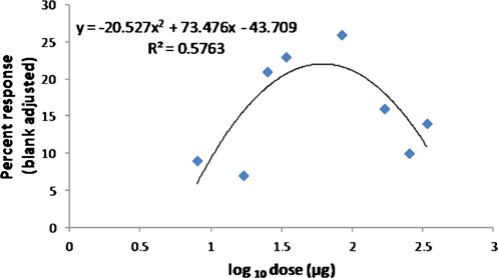
Percentage of *Culex pipiens* flying upwind in a dual-choice flight olfactometer in response to different concentrations of a three-component kairomone blend (benzaldehyde, (*E*)*-2-*nonenal, and phenylacetaldehyde) after subtracting the response to a paired solvent (pentane) control

Because quantity as well as quality can determine the intensity of chemically mediated behavior, *C. pipiens* response was measured in the flight olfactometer to different doses of the three-component blend compared to a solvent control. There was a significant positive quadratic response [*log*(dose) (y = –20.527x^2^ + 73.476x–43.709; *R* ^2^ = 57.6 %], with a response maximum predicted at 62 μg (Fig. 5). The response flattened out at both the lowest and highest doses; removal of the two most extreme doses produced a stronger-fitting regression model (*R* ^2^ = 93.3 %), but with a similar predicted optimal dose of 65 μg.

## Discussion

A positive upwind orientation by *C. pipiens* to floret volatiles of *A. syriaca* was demonstrated in this study, suggesting a new potential source of attractants for use in the field as lures. Males and females showed significant response to milkweed florets, a pentane extract, and a synthetic blend of the extract’s six major constituents: benzaldehyde, (*E*)-β-ocimene, benzyl alcohol, phenylacetaldehyde, nonanal, and (*E*)-2-nonenal. The first three of these compounds are among the most common floral odor constituents in plants (Knudsen et al., 2006). Subsequent subtractive bioassays of the synthetic blend demonstrated that only three of its components contributed to attraction: benzaldehyde, phenylacetaldehyde, and (*E*)-2-nonenal.

The attraction of mosquitoes to plant odors has been demonstrated in numerous field and laboratory studies, but few studies have shown significant attraction to synthetics. Mauer and Rowley (1999) observed orientation of *C. pipiens* to a methylene chloride extract of *A. syriaca* in a dual port olfactometer. However, a synthetic blend of the two dominant compounds in the extract headspace, benzyl alcohol and 2-phenylethanol, failed to attract the mosquitoes. We did not test 2-phenylethanol, but benzyl alcohol was not active in our flight olfactometer bioassays. Jhumur et al. (2007) also found benzyl alcohol to not be very attractive to *C. pipiens* var. *molestus*, although Puri et al. (2006) recorded significant upwind response by *C. quinquefasciatus* to the compound.

Flowers of *Tanacetum vulgare* also attract *Culex* species in the field (Andersson and Jaenson, 1987), and Bowen (1992b) discovered a high proportion of both broadly- and narrowly-tuned antennal receptor neurons of *C. pipiens* sensitive to thujone, the primary constituent of *T. vulgare* fragrance. However, thujone only elicited a close-range dose-dependent probing response in *C. pipiens*; it did not stimulate upwind flight in a wind-tunnel olfactometer (Bowen, 1992b). The only previous flower-based synthetic blend causing mosquito orientation was developed from *Silene otites*, whose flowers attract mosquitoes in the field (Brantjes and Leemans, 1976). The synthetic blend mimic consisted of phenylacetaldehyde, veratrole, and 2-methoxyphenol, the first of which elicited the strongest attraction in *C. pipiens* var. *molestus* when presented individually (Jhumur et al., 2006). Phenylacetaldehyde also was a prominent component of our *A. syriaca* headspace and solvent extract, and it was essential for maximum attraction to the synthetic blend.

Although nonanal was a major component of *A. syriaca* floret extract, we saw no evidence that it played a role in attraction in our bioassays. In contrast, from single-cell recordings, Syed and Leal (2009) determined that ca. 40 % of all antennal olfactory receptors of *C. quinquefasciatus* were acutely sensitive to nonanal, and it produced a synergistic effect on trap catch when presented with CO_2_. This apparent discrepancy likely is explained by differences in mosquito physiological state. Nonanal is a major skin odorant of birds and mammals, including humans, resulting from the oxidation of sebaceous fatty acids (Haze et al., 2001). This fact, along with the significant behavioral interaction with CO_2_, suggests that nonanal may play an important role in mediating *Culex* host-finding, but does not attract sugar-seeking mosquitoes.

We found notable differences between profiles of the pentane extract and the SPME-collected headspace of *A. syriaca* florets. Pentane extracts were dominated by phenylacetaldehyde, (*E*)-β-ocimene, and nonanal, whereas the headspace profile contained primarily benzaldehyde, ocimene isomers, and phenylacetaldehyde. Disparities between extracts and chemicals collected in the headspace might be explained by differences in the physico-chemical properties of the constituents, deep penetration by solvents to extract compounds that are not normally released by the tissue, and/or selective trapping of chemicals by SPME. In this study, differences between the methods are largely consistent with chemical differences in vapor pressure. Benzaldehyde, β-ocimene, and alloocimene have the highest vapor pressures of all the chemicals identified from milkweed, and they make up most of the volatile profile. The vapor pressure of benzaldehyde is almost twice that of nonanal, almost four times that of (*E*)-2-nonenal, and more than six times that of benzyl alcohol. The latter three compounds were either absent or found in very low levels in the headspace analysis. Phenylacetaldehyde is intermediate in its volatility, but was also the component found in the highest levels in the pentane extract.

Volatile collection analyses can produce misleading results, as they may not accurately reflect the actual proportionality of constituents in the headspace. We do not believe that this was a major factor for explaining the differences in the headspace and extract profiles of milkweed due to our choice of SPME phase. Although PDMS has been the most widely used SPME phase, it is actually a poor choice for characterizing plant volatile profiles that contain constituents with a range of volatilities and functional groups. In preliminary studies (not shown), we found a broader array of volatile compounds was trapped from various flowers by DVB/CAR/PDMS compared to PDMS alone. This mixed-bed fiber not only employs a broader range of phase polarity, but also incorporates both adsorption and partitioning as mechanisms of collection (Koziel and Novak, 2002). A number of recent studies have demonstrated the higher recovery efficiency and linearity by DVB/CAR/PDMS compared to other SPME phases for all of the compound classes that we identified from milkweed (Cui et al., 2009; Ferreira et al., 2009; Zhang et al., 2009). For example, Zhang et al. (2009) found that volatile profiles of longan fruit extracted by a 50/30 μm DVB/CAR/PDMS fiber were 3x higher in terpenes, 5x higher in alcohols, and 14x higher in esters than those produced by a 100 μm PDMS fiber, and revealed volatile carbonyls and acids, which were completely absent from PDMS profiles.

This study points to floral odors as a potential new model for chemical lures useful for mosquito sampling or control. If similar attraction can be demonstrated at the field level, as has already been demonstrated with human kairomone blends (Mukabana et al., 2012), synthetic floral blends could potentially be used in trapping devices to sample adult populations. Relative to animal-derived odors (Mukabana et al., 2012), floral odors have the advantage of attracting both sexes of mosquitoes in proportional numbers, and females in all gonotrophic states and in reproductive diapause. Given that *C. pipiens* visits a variety of flowers for nectar-feeding, the three compounds identified here are likely not the only floral components attractive to them, and more effective blends may remain to be discovered. It also remains to be seen whether different mosquito species use similar chemical cues. Future research should seek additional attractants and determine optimal blend release rates, delivery systems, and trap designs for maximizing capture in the field.
